# Awareness and Willingness towards Organ Donation among Riyadh Residents: A Cross-Sectional Study

**DOI:** 10.3390/healthcare12141422

**Published:** 2024-07-16

**Authors:** Baraa Alghalyini, Abdul Rehman Zia Zaidi, Zainudheen Faroog, Mohammad Salman Khan, Saad Rahman Ambia, Golam Mahamud, Hala Tamim

**Affiliations:** 1 Department of Family & Community Medicine, College of Medicine, Alfaisal University, Riyadh 11533, Saudi Arabia; balghalyini@alfaisal.edu (B.A.); htamim@yorku.ca (H.T.); 2 College of Medicine, Alfaisal University, Riyadh 11533, Saudi Arabia; zfaroog@alfaisal.edu (Z.F.); mokhan@alfaisal.edu (M.S.K.); sambia@alfaisal.edu (S.R.A.); gmahamud@alfaisal.edu (G.M.)

**Keywords:** organ donation, Saudi Arabia, health surveys, knowledge, attitudes

## Abstract

Background: The increasing prevalence of chronic diseases in Saudi Arabia has heightened the need for organ transplantation; however, the donor pool remains insufficient. This study explored awareness and willingness towards organ donation among Riyadh residents and examined the sociodemographic factors influencing these attitudes. Methods: A cross-sectional survey using convenience sampling was conducted among adults in Riyadh. The survey assessed demographic characteristics, awareness, willingness to donate, and sociodemographic factors. Statistical analyses included descriptive statistics and logistic regression. Results: Among the 645 respondents, 56.4% were willing to donate organs, with females showing a higher propensity than males (OR 2.9, 95% CI 1.7–5.1, *p* < 0.001). Awareness of organ donation centers was linked to increased willingness to donate (OR 1.5, 95% CI 1.1–2.5, *p* < 0.001). Higher educational level was strongly associated with donor registration (OR 36.8, 95% CI 14.7–91.9, *p* < 0.001). Despite their high willingness, only 9.5% were registered as donors, highlighting the gap between intention and action. Conclusions: Riyadh residents showed a significant willingness to donate organs, influenced by gender, education, and awareness. Low registration rates suggest barriers such as religious beliefs and lack of information. Targeted educational campaigns and policy evaluations, including an opt-out system, are recommended to enhance registration rates.

## 1. Introduction

Organ transplantation is the pinnacle of modern medical interventions that offers a critical lifeline for patients with end-stage organ failure, yet the disparity between organ supply and demand presents a global health challenge, particularly in regions with high rates of chronic conditions [1]. In Saudi Arabia, the increasing prevalence of such diseases accentuates the need for organ transplants, highlighting the importance of organ donation [2].

Societal awareness and willingness to donate are essential for expanding the pool of organ donors. Various studies have highlighted the significant influence of sociocultural, religious, and educational factors on individuals’ attitudes towards organ donation [3,4]. Despite potential cultural support for organ donation in Saudi Arabia, actual donation rates remain low, with only 3.77 donors per million people, far below the need [5].

This study investigated organ donation awareness and willingness among residents of Riyadh, the capital of Saudi Arabia, by analyzing the sociodemographic factors that shape these attitudes. This research aims to contribute to existing knowledge and inform targeted interventions to boost regional organ donation rates. This study emphasizes the need for enhanced public education and awareness to address misconceptions and foster a supportive environment for organ donation. This aligns with previous scholarly endeavors that have highlighted the critical need for enhanced public education and awareness to address prevalent misconceptions and cultivate a more organ donation-conducive environment [6,7].

By examining public awareness, attitudes, and sociodemographic influences, this study sought to quantify awareness and willingness levels and explore religious, cultural, and ethical factors that may affect organ donation decisions. Aligning public attitudes with healthcare needs is vital for increasing donor rates and improving health outcomes for those requiring transplantation.

## 2. Materials and Methods

### 2.1. Study Design

This observational cross-sectional study aimed to measure awareness, attitudes, and willingness towards organ donation in the Saudi population from April 2023 to December 2023. The survey also assessed the registration rate of potential deceased organ donors.

### 2.2. Sampling, Participants, and Data Collection

Data were collected through in-person solicitations in various public areas, including malls, public parks, restaurants, and cafés. An anonymous online survey was also conducted via social media platforms (WhatsApp, Facebook, and Twitter) using the snowball technique. Convenience sampling was employed to recruit the participants. Electronic informed consent was obtained before participation, with an introductory consent form outlining the study’s objectives and ethical considerations and assuring participants of the anonymity and voluntary nature of their participation. The inclusion criteria were adults aged over 18 years residing in Riyadh during the data collection phase.

### 2.3. The Survey Instrument

A structured questionnaire was developed after an extensive literature review of the topic [3,6,8,9,10,11]. To ensure content validity, the questionnaire was reviewed by a panel of experts, including transplantation consultants, organ donation program directors from King Faisal Specialist Hospital, and specialists in family and community medicine and public health. Additionally, a pilot study involving 20 participants from the target demographic group was conducted to evaluate the clarity and average completion time of the questionnaire. Feedback from this phase led to refinements, including an Arabic version, to accommodate language preferences. The questionnaire consisted of three sections: (1) demographic factors (education level, age, gender); (2) willingness to donate an organ and registration as a potential deceased organ donor; and (3) awareness (knowledge of organ donation, perceptions of its importance, incentives and barriers to registration, and awareness of organ donation facilities in Saudi Arabia).

### 2.4. Sample Calculation

The sample size (*n*) was determined using Cochran’s Sample Size Formula with the assumption of a 95% confidence level (*Z*  =  1.96); e is the margin of error, which was 5%; *p* is the (estimated) proportion of the population that has the attribute in question, which equaled 50% (or 0.5); *q* is 1 − *p*:

*n*_0_ = *Z*^2^*pq*/*e*^2^

After applying this formula, the required sample size (*n*) was 385. The target sample size was increased to 645 to account for potential nonresponses and incomplete surveys, ensuring a strong and reliable dataset.

### 2.5. Statistical Data Analysis

The collected data were encoded in Microsoft Excel and cleaned to remove missing data. Data analysis was performed using SPSS version 26 (Statistical Package for the Social Sciences, IBM Corp., Armonk, NY, USA). Descriptive statistics (frequencies, percentages, means, and standard deviations) were used to summarize the participant demographics and responses.

Multiple-response dichotomy analysis was applied to the questions with multiple responses. Multivariable logistic binary regression analysis (MLBR) was conducted to identify statistically significant predictors of organ donation registration, awareness of organ donation regulations, and awareness of the Saudi National Center for Organ Transplant (SCOT) among the participants. MLBR outcomes were reported as odds ratios (ORs) with 95% confidence intervals (CIs). A *p*-value of <0.05 was considered indicative of statistical significance.

### 2.6. Ethical Considerations

Ethical approval was granted by the Institutional Review Board of Alfaisal University (IRB-20219). The study adhered to ethical standards, ensuring participant confidentiality and the right to withdraw without any consequences. Informed consent was obtained electronically, with an introduction explaining the study’s purpose, confidentiality measures, and voluntary nature of participation.

## 3. Results

A total of 645 participants from various districts of Riyadh completed the survey. Thirty-three participants were excluded because of missing data, resulting in a final sample size of 612 participants.

### 3.1. Sociodemographic Characteristics

The most common age group was 21–30 years (68.1%), followed by those aged ≤20 years (26.0%) and >30 years (10.0%). Of the respondents, 63.4% were female (*n* = 388), and 36.6% were male (*n* = 224). The majority had an education level lower than high school (77.0%), and 57.5% were Saudi nationals.

### 3.2. Organ Donation Registration and Willingness

Only 9.5% (*n* = 58) of the participants were registered as organ donors, while 56.4% (*n* = 345) expressed willingness to donate their organs. At the bivariate level, factors significantly associated with being registered as an organ donor included being male, older age, Saudi nationality, having education beyond high school, and awareness of the existence of a center for organ donation (Table 1). Conversely, willingness to donate an organ was significantly associated with being female, having an education beyond high school, and being aware of the existence of a center for organ donation.

### 3.3. Reasons for Willingness and Unwillingness

Among those willing to donate (*n* = 345), 63% were willing to be deceased donors. Reasons for willingness included the desire to help others (85.2%) and religious motives (14.2%). Among those unwilling to donate (*n* = 267), the primary reason was religious beliefs (80.5%), followed by the fear of not receiving appropriate medical care (8.2%) (Table 2).

### 3.4. Factors Influencing Organ Donation Registration

Multivariable logistic regression analysis revealed that participants aged 21–30 years were less likely to be registered as organ donors than those aged ≤20 years (OR 0.3, 95% CI 0.1–0.7, *p* = 0.009). Saudi nationals were more likely to be registered as organ donors than non-Saudis (OR 2.4, 95% CI 1.0–5.6, *p* = 0.045). Participants with education beyond high school were significantly more likely to be registered as organ donors than those with lower education levels (OR 36.8, 95% CI 14.7–91.9, *p* < 0.001). Awareness of organ donation centers was also a significant predictor of organ donation registration (OR 10.7, 95% CI 4.4–26.0, *p* < 0.001) (Table 3).

### 3.5. Factors Influencing Willingness to Donate

Willingness to donate organs was significantly associated with being female (OR 2.9, 95% CI 1.7–5.1, *p* < 0.001), having education beyond high school (OR 1.5, 95% CI 1.0–2.2, *p* = 0.043), and awareness of organ donation centers (OR 1.5, 95% CI 1.1–2.5, *p* < 0.001).

In addition, participants who were aware of organ donation centers were 10.7 times more likely to be registered as organ donors than those who were unaware of organ donation centers when sociodemographic variables were held constant (OR = 10.7, *p* < 0.001). On the other hand, for willingness to participate in organ donation, males were at reduced odds of being willing to donate organs (OR = 1.2, *p* = 0.704), whereas participants who were aware of organ donation centers showed significantly increased odds of donating organs OR (95% CI) = 1.5 (1.1–2.5).

This study highlights a significant willingness among Riyadh residents to donate organs, influenced by gender, education level, and awareness of organ donation centers. However, actual registration rates remain low, indicating potential barriers such as religious beliefs and lack of information. Addressing these issues through targeted educational and awareness campaigns could enhance registration rates and support the implementation of an opt-out system for organ donation in Saudi Arabia.

## 4. Discussion

This study aimed to investigate the awareness of and willingness toward donating organs among residents of Riyadh, Saudi Arabia, and to identify the sociodemographic factors influencing these attitudes. These findings highlight several important aspects that can inform future interventions and policy changes. Organ donation can save the lives of those in need of transplantation, which is a crucial problem in contemporary health care. Similar to many other nations, Saudi Arabia is experiencing a scarcity of organ donors, resulting in lengthy waiting lists for transplant recipients [1]. Increasing the rate of organ donation registration is one way to increase the number of organ donors.

### 4.1. Factors Associated with Willingness to Donate Organs

The results indicate that females were more willing to donate organs than males, consistent with previous studies in Saudi Arabia and other regions [6,12,13]. A study in India showed that female donors constituted 71% of the living-donor pool [14].

This could be explained by two factors. First, women may exhibit higher levels of empathy and altruism, which may explain why they are more likely to donate organs. Organ donation is strongly influenced by altruistic motives such as the desire to aid others or advance societal welfare. In addition, 85.2% of study participants’ main reason to donate organs was the desire to help people, and 68.8% of participants said they would be prepared to donate their organs to anyone, not just family members who support and reflect altruistic behavior. Second, women are often perceived as caregivers and nurturers of families and communities. Due to this emphasis on interpersonal relationships, women may view organ donation as a means of improving the lives of others and fulfilling their responsibilities as caregivers and contributors to the well-being of their families. Therefore, the decision to donate among women is highly influenced by the donor–recipient relationship. A study examining sex disparities in organ donation and transplantation conducted in India showed that female donors were more frequently mothers (33.7%) and wives (20.1%) than organ recipients [14].

Understanding these gender differences is crucial in designing targeted awareness campaigns that address the specific concerns and motivations of different demographic groups. To create a more inclusive and knowledgeable society regarding organ donation, transplant centers should consider the many motives and viewpoints of people regardless of gender. A deeper re-evaluation of traditional gender roles and a woman’s function in her family is necessary to address the gender imbalance.

There was a significant association between awareness of organ donation facilities, such as the Saudi Center for Organ Transplantation (SCOT), and willingness to donate. A similar study conducted in Saudi Arabia showed that participants had strong beliefs about the importance of organ donation, with those holding this belief more than three times as likely to be aware of SCOT [15]. This can be ascribed to the fact that these facilities play a role in dispelling myths surrounding organ donation. People can better comprehend the structured and moral framework surrounding organ donation when they are aware of the presence, roles, and effects of specialized centers such as SCOT. These facilities run awareness initiatives that help to clarify misconceptions, address cultural and religious issues, and provide accurate information about the entire donation process. Consequently, people gain more knowledge, have more faith in the system, and are more inclined to view organ donation as a respectable and beneficial way of improving others’ lives. Furthermore, the centers provide information about incentives offered by the government, which are not readily available on the Internet, such as the third-degree King Abdulaziz Medal to all donors, 50% discounts on national flights, and 50,000 Saudi riyals given in full immediately after the donation. Moreover, the government provides access cards to donors as part of a donor incentive program to facilitate banking and travel services. People who donate their organs also have full-time access to healthcare facilities for checkups and health maintenance [16].

This study also showed that the participants were aware of when someone could donate their organs. Among the participants, 36.5% were willing to give up organs after they passed away. Furthermore, 27% believed that the time of donations made during life or after death was the same. This contrasts with a similar study conducted in Jazan, Saudi Arabia, which found that 58% of participants were willing to donate after death and 34% saw that there was no difference in the timing of donation, whether during life or after death [17]. In addition, a study conducted by Al-Kharj showed that 55.6% of the participants would donate after death [8]. However, a study conducted in eastern Morocco found that more than 90% of the study participants knew that organ donations can come from both cadaveric and living persons [18] and that the disparity in the time of organ donation knowledge varies depending on the person’s culture, educational background, access to healthcare, and involvement in campaigns.

### 4.2. Barriers to Organ Donation

Although the study participants had a positive attitude towards organ donation, 43.6% of them were not willing to donate organs. Similar studies conducted in Saudi Arabia showed that approximately 43.3% and 33.9% of participants refused organ donation [11,19]. In contrast, a study conducted in Jazan showed that only 5.5% of study participants exhibited a negative attitude towards organ donation [17]. A significant barrier to organ donation identified in this study was religious beliefs, with 80.5% of those unwilling to donate citing this reason. The percentage in the present study was eight times higher than the percentage of unwillingness in the Jazan study.

This value was higher than that reported in other studies conducted in Saudi Arabia. A study conducted among Saudis in Madinah City showed that 21.7% of participants lacked awareness of organ donation and 6.8% cited religion as the primary reason for refusing organ donation [10]. In a previous study conducted among the public in Saudi Arabia, one of the most common barriers to organ donation was religious reasons (47.8%) [20]. Similarly, research conducted at the Dhahran Military Hospital revealed that 26.2% believed organ donation was banned in Islamic tradition [20].

Islam plays a significant role, as Saudi society is deeply rooted in Islamic values, which may influence perceptions of organ donation. The predominantly Muslim Saudi society’s attitudes towards organ donation are significantly shaped by Islamic jurisprudence, which also governs legal considerations of brain death and organ transplantation. Despite the fatwa that organ donation saves hundreds of lives and helps others, which is consistent with Islamic teachings on helping others and the Holy Quran and Sunnah, it is explicitly stated that Muslims should preserve their lives and help each other [21,22]. The reason for the high percentage of religion as a barrier cannot be extrapolated from this study’s findings.

### 4.3. Factors Associated with Organ Registration

This is one of the first studies to explore the actual public registration rates for potential deceased organ donors and to investigate the sociodemographic factors associated with the organ-registered status of the participants. This study found that only 9.5% of participants were registered as organ donors; this is lower than another study conducted in Saudi Arabia, which showed 21.2% registration as potential organ donors via the Tawakkalna app [15]. The results of this study highlight four important factors that are associated with registration.

First, younger participants, aged 20–30 years, were less likely to be registered as organ donors. A similar study conducted in Saudi Arabia showed that individuals aged 45 years or older were less likely to register (OR 0.894, *p*-value = 0.031) [15]. One possible reason for this could be that the younger generation may not be aware of the significance of organ donation and its potential to save lives. This demographic group may benefit greatly from educational programs aimed at closing this knowledge gap. Furthermore, cultural and religious factors can come into play because younger people might not be completely aware of how organ donation aligns with Islamic teachings. This finding aligns with the evidence from the SCOT Annual Report 2020, in which the age distribution of deceased donors showed that 14% were ≤20 years old, 24% were 21–30 years old, and 62% were >30 years old [23].

Second, Saudi nationals were 2 times more likely to be registered as organ donors. A similar study showed the same trend, where Saudi citizens were significantly more likely to register than expatriates, showing more than twice the likelihood (OR 2.33, *p* < 0.001) [15]. In addition, the demographic characteristics of the annual SCOT report showed that 70% of donors were Saudi nationals, while 30% were non-Saudi [23].

The lower registration rate among foreigners may be due to the logistical difficulties in reaching and teaching diverse expatriate populations in Saudi Arabia. Implementing inclusive awareness initiatives, resolving cultural differences, and overcoming language hurdles could be crucial steps in encouraging organ donation registration among expatriate communities in Saudi Arabia. As organs are scarce, and the demand for transplantation is increasing, it is important to include a diversified organ pool and utilize everyone in society to improve the community, irrespective of nationality.

Finally, participants with an education higher than high school were 10 times more likely and participants who were aware of SCOT were 36 times more likely to be registered as organ donors. As the study was conducted in the capital of the country and Riyadh is considered the most developed city, it can be expected that people living in cities will place themselves in closer proximity to medical facilities and increase their awareness of the educational programs offered by these establishments. Considering this, it is anticipated that they are more knowledgeable about the medical services offered than those in rural areas in the country. Furthermore, transplant centers provide people with a thorough understanding of the vital role that these establishments play in organizing the processes involved in organ donation and transplantation. This information probably allays any doubts, anxieties, or misconceptions about organ donation, nurturing a favorable view of the procedure.

### 4.4. Policy Implication

Despite the positive attitudes and willingness of participants, the actual number of registered organ donors tended to be lower in this study. Another study followed a similar trend, in which 78.8% had not registered and 89.2% were willing to donate all organs [15]. A considerable bias existed between the two domains. Therefore, the authors believe that the government should consider moving towards an opt-out donation policy in which all individuals residing in a country/state are willing deceased organ donors unless they specifically opt out of doing so. This is also known as “presumed consent’” [24]. In this system, declining to donate an organ using an app or another formal platform is a legally binding declaration of a person’s desire to not donate an organ. Assuming that people are willing donors, unless they express different opinions, this strategy seeks to increase the rate of organ donation. A recent PROSPERO-registered systematic review considered the differences in transplant numbers between opt-in and opt-out countries and concluded that opt-out countries boasted demonstrably greater deceased donor rates than opt-in countries [25]. Spain is another country that considers its achievements in the transplant arena due to its opt-out policy. Spain has the highest number of deceased donors PMP internationally, 49.6 in 2019, compared to 36.8 in the USA in 2019 [26].

Therefore, the authors believe that it is important to conduct research on the public’s opinions and concerns regarding an opt-out policy with cultural, ethical, and legal considerations. If an opt-out policy is implemented, the authors believe that appropriate campaigns can increase the organ pool and shorten waiting times in the transplantation pool.

### 4.5. Practical Implications and Social Contributions

Increasing organ donation rates can have profound impacts on public health by reducing waiting times for transplants and improving patient outcomes. Awareness campaigns should focus on the altruistic benefits of organ donation, highlighting personal stories and testimonials that resonate with potential donors. Additionally, providing incentives and recognizing donors through programs such as the King Abdulaziz Medal can further encourage participation.

### 4.6. Strengths and Limitations

A notable strength of this study is its comprehensive assessment of attitudes and behaviors toward organ donation among a diverse population in Riyadh utilizing a well-structured questionnaire that covers various sociodemographic factors, including gender, age, education, and awareness of organ donation centers. The inclusion of a broad range of participants enhances the generalizability of the findings within the urban Riyadh context.

However, this study had some limitations that must be acknowledged. First, the cross-sectional design precludes the inference of causal relationships between the identified factors and willingness or registration for organ donation. Second, the study’s reliance on self-reported data might have introduced response biases, potentially affecting the accuracy of the reported attitudes and behaviors. Additionally, the focus of this study on the urban population of Riyadh may limit the applicability of the findings to rural or other urban areas in Saudi Arabia, where cultural and societal norms regarding organ donation may differ. Lastly, we could have collected data from more diverse areas like government offices, construction sites, grocery stores, bus stops, sports events, or other venues. Future studies could address these limitations by employing longitudinal designs, incorporating objective measures of organ donation behavior, and expanding the sample to include a more diverse range of geographical settings.

## 5. Conclusions

This cross-sectional study, conducted among residents of Riyadh, Saudi Arabia, highlights a significant willingness to engage in organ donation, influenced by gender, education, and awareness of organ donation centers. The findings revealed that females are more likely to donate organs than males, driven by higher levels of empathy and altruism. Education and awareness play crucial roles, and higher educational levels and knowledge of the Saudi Center for Organ Transplantation (SCOT) are strongly associated with increased registration as organ donors. Despite the high willingness to donate, actual registration rates remain low, indicating potential barriers, such as religious beliefs and lack of information. Addressing these barriers through targeted educational campaigns that engage religious leaders and provide accurate information on organ donation can enhance public understanding and participation. Policy interventions such as implementing an opt-out system in which individuals are presumed willing to donate unless they opt out could significantly increase donor rates. Evidence from other countries with opt-out systems suggests higher donor rates compared to opt-in systems. Future research should explore public opinion on such policy changes and consider the cultural, ethical, and legal factors specific to Saudi Arabia.

In conclusion, enhancing organ donation rates in Saudi Arabia requires a multifaceted approach that includes targeted education, policy changes, and addressing cultural and religious barriers. These efforts can help bridge the gap between willingness and actual registration, ultimately improving health outcomes for patients in need of organ transplantation.

## Figures and Tables

**Table 1:** Bivariate relationship between organ donation and sociodemographic and registered status as organ donor factors among study participants.

	Total	Registered as Organ Donor			Willing to Donate Organ		
	*n* (%)	%	OR (95% CI)	*p*-Value	%	OR (95% CI)	*p*-Value
**Sociodemographic Characteristics**							
**Gender**							
Females	388 (63.4)	5.9	1		63.1	1	
Males	224 (36.6)	15.6	2.9 (1.7–5.1)	**<0.001**	44.6	0.5 (0.3–0.7)	**<0.001**
**Age**							
≤20	159 (26.0)	17.0	1		53.5	1	
21–30	392 (64.1)	3.1	0.2 (0.1–0.3)	**<0.001**	57.4	1.2 (0.8–1.7)	0.399
>30	61 (10.0)	31.1	2.2 (1.1–4.4)	**0.023**	57.4	1.2 (0.6–2.1)	0.602
**Nationality**							
Saudi	352 (57.5)	15.8	3.7 (2.0–6.7)		56.9	0.9 (0.7–1.3)	0.813
Non-Saudi	260 (42.2)	4.8	1	**<0.001**	56.0	1	
**Education**							
Less than high school	471 (77.0)	1.5	1		54.1	1	
Above high school	141 (23.0)	36.2	37.7 (16.5–85.4)	**<0.001**	63.8	1.5 (1.0–2.2)	**0.043**
**Aware of Center for Organ Donation**							
Yes	315 (51.5)	15.6	5.9 (2.8–12.2)	**<0.001**	43.8	0.3 (0.2–0.5)	**<0.001**
No	297 (48.5)	3.0	1		69.7	1	
**Registered as an Organ Donor**							
Yes	58 (9.5)						
No	554 (90.5)						
**Willing to Donate Organ**							
Yes	345 (56.4)						
No	267 (43.6)						

**Bold** values indicate statistically significant results at *p* < 0.05.

**Table 2:** Participants’ attitudes toward and reasons for donating organs.

Question	Factor	Percentage (%)
When would you like to donate your organs?	After death	36.5
	During lifetime	36.5
	Both	27
To whom are you willing to donate your organs?	Relatives	32.2
	Both nonrelatives and relatives	68.8
What is your prime reason?	Religious motive	14.2
	Social motive	0.6
	Desire to help	85.2
If you are not willing to donate your organs, what are the reasons?	Family and social barriers	3
	Religious belief	80.5
	Fear of not receiving appropriate medical care	8.2

**Table 3:** The results of multivariable logistic regression for the association between organ donation and sociodemographic characteristics and registered status as organ donors among the study participants.

	Registered as Organ Donor		Willing to Donate Organ	
	Adjusted OR (95%CI)	*p*-Value	Adjusted OR (95%CI)	*p*-Value
**Sociodemographic Characteristics**				
**Gender**				
Females	1		1	
Males	1.2 (0.5–2.8)	0.704	0.5 (0.4–0.8)	**<0.001**
**Age** (years)				
18–20	1		1	
21–30	0.3 (0.1–0.7)	**0.009**	1.1 (0.7–1.6)	0.645
>30	1.3 (0.5–3.3)	0.595	1.6 (0.8–3.1)	0.146
**Nationality**				
Saudi	2.4 (1.0–5.6)	**0.045**	0.9 (0.6–1.3)	0.463
Non-Saudi	1		1	
**Education**				
Less than high school	1		1	
Above high school	36.8 (14.7–91.9)	**<0.001**	1.5 (1.0–2.3)	0.073
**Aware of Center for Organ Donation**				
Yes	10.7 (4.4–26.0)	**<0.001**	1.5 (1.1–2.5)	**<0.001**
No	1		1	

**Bold** values indicate statistically significant results at *p* < 0.05.

## Data Availability

The data used to support the findings of the study are available upon request from the corresponding author.
